# Quality of Life and Mental Health Status of Arsenic-affected Patients in a Bangladeshi Population

**DOI:** 10.3329/jhpn.v30i3.12289

**Published:** 2012-09

**Authors:** Emdadul H. Syed, Krishna C. Poudel, Kayako Sakisaka, Junko Yasuoka, Habibul Ahsan, Masamine Jimba

**Affiliations:** ^1^Department of Community and Global Health, Graduate School of Medicine, The University of Tokyo, 7-3-1 Hongo, Bunkyo-ku, Tokyo 113-0033, Japan; ^2^Department of Health Studies, University of Chicago, 5841 South Maryland Avenue, Suite N102, Chicago, IL 60637, USA

**Keywords:** Arsenic, Arsenic contamination, Cross-sectional study, Mental health, Quality of life, Whoqol-Bref, Bangladesh

## Abstract

Contamination of groundwater by inorganic arsenic is one of the major public-health problems in Bangladesh. This cross-sectional study was conducted (a) to evaluate the quality of life (QOL) and mental health status of arsenic-affected patients and (b) to identify the factors associated with the QOL. Of 1,456 individuals, 521 (35.78%) were selected as case and control participants, using a systematic random-sampling method. The selection criteria for cases (n=259) included presence of at least one of the following: melanosis, leucomelanosis on at least 10% of the body, or keratosis on the hands or feet. Control (non-patient) participants (n=262) were selected from the same villages by matching age (±5 years) and gender. The Bangladeshi version of the WHOQOL-BREF was used for assessing the QOL, and the self-reporting questionnaire (SRQ) was used for assessing the general mental health status. Data were analyzed using Student's *t*-test and analysis of covariance (ANCOVA), and the WHOQOL-BREF and SRQ scores between the patients and the non-patients were compared. The mean scores of QOL were significantly lower in the patients than those in the non-patients of both the sexes. Moreover, the mental health status of the arsenic-affected patients (mean score for males=8.4 and females=10.3) showed greater disturbances than those of the non-patients (mean score for males=5.2 and females=6.1) of both the sexes. The results of multiple regression analysis revealed that the factors potentially contributing to the lower QOL scores included: being an arsenic-affected patient, having lower age, and having lower annual income. Based on the findings, it is concluded that the QOL and mental health status of the arsenic-affected patients were significantly lower than those of the non-patients in Bangladesh. Appropriate interventions are necessary to improve the well-being of the patients.

## INTRODUCTION

Arsenic is a metalloid element widely distributed in water, air, and soil (1,2). It was found in groundwater in many countries and is highly toxic for humans (3). Results of epidemiological studies indicate that chronic exposure to arsenic increases the risk of arsenic-induced diseases (4-6). Arsenic may also cause low birthweight and many physical and neurological deficiencies (7).

Contamination of groundwater by inorganic arsenic is one of the major public-health hazards in Bangladesh (8,9). According to the World Health Organization (WHO), the problem is “the largest mass poisoning of a population in history” (10). In the beginning of the 1970s, about 10 million hand-pump wells were installed for the prevention of waterborne diseases in Bangladesh (11). However, natural contamination of groundwater by arsenic was realized only in the late 1990s in these wells. In Bangladesh, an estimated 35-77 million people have been chronically exposed to arsenic through drinking-water (12). As a result, 18.7% (n=140) of the study participants (n=750) reported at least one arsenic-affected patient in their households (13).

Arsenic-affected patients have been facing many problems in their daily life. From a social perspective, arsenic-affected people are barred from community activities in Bangladesh (14). For example, women with visible symptoms of arsenicosis may not be able to find marriage partners, and husbands sometimes divorce the affected wives (14). Children with symptoms of arsenicosis also face the prejudice of their peers and may attempt to hide their symptoms when attending school (15).

Under such conditions, the quality of life (QOL) and mental health status of arsenic-affected patients might be of concern. Results of a study showed that arsenic-affected patients had lower QOL than those of controls in Bangladesh (16). However, the study was conducted in four villages, with only 104 patients. Besides the QOL, the burden of mental health problems was significantly higher among people living in arsenic-exposed villages (54%) compared to those in arsenic-free villages (34%) in Inner Mongolia, China (17). In the Wisconsin counties in the USA, arsenic-affected people are known to suffer from depression (18).

The assessment of QOL and mental health status of arsenic-affected patients is important as arsenic-induced suffering has strong social and psychological implications. On the other hand, we need more research on the QOL and mental health status of arsenic-affected patients. To the best of our knowledge, no study was conducted on both QOL and mental health status among the same arsenic-affected patients. Therefore, in this study, we aimed (a) to determine the QOL and mental health status of arsenic-affected patients compared to those of non-patients and (b) to identify the factors associated with the QOL of arsenic-affected people in Bangladesh.

## MATERIALS AND METHODS

### Study site

This cross-sectional study was conducted in Araihazar upazila of Narayanganj district in Bangladesh. Araihazar upazila is located 25 km southeast from the capital city Dhaka. The total area of upazila is 183.35 square km, and its population is about 300,000 (19). Most people drink water from tubewells containing arsenic concentrations ranging from <10 µg/L to 864 µg/L (20); the arsenic concentration of each tubewell is labelled by the Joint Arsenic and Health Research Project of the Columbia University and the University of Chicago to set arsenic levels that are not hazardous. Of 6,000 tubewells in the study area, 60% had arsenic concentrations of >50 µg/L (20). Of 70,000 people in the study area, about 22% were exposed to the levels of 101 300 µg/L arsenic concentration (20). Briefly, arsenic concentrations of tubewell-water were measured using graphite furnace atomic absorption spectrometry, with a detection limit of 5.0 µg/L. Samples below the limit of detection were subsequently re-analyzed using inductively-coupled plasma-mass spectrometry, with a detection limit of 0.1 µg/L (21). Details of measurements of the arsenic exposure were reported earlier (22). The national standard for arsenic in drinking-water in Bangladesh is ≤50 µg/L; according to the guideline of the World Health Organization (WHO), the standard for arsenic in drinking-water is ≤10 µg/L (21).

A prospective cohort study titled “The health effects of arsenic longitudinal study” (HEALS) (20) was conducted in 2000 in Bangladesh by the Joint Arsenic and Health Research Project of the Columbia University and the University of Chicago. We targeted the participants of this cohort for our study.

### Participants

We collected data from 521 participants in 11 villages of the HEALS study area in Bangladesh. In total, 1,456 individuals (patients=813 and non-patients=643) from those villages were included in the database of the ongoing HEALS study. Of these individuals, we selected 540 participants who met the inclusion criteria of the study. Of the 540 participants, we collected data from 521 participants (259 arsenic patients and 262 non-patients). The inclusion criteria for the patients were: a history of drinking arsenic-contaminated water (>50 µg/L) and the presence of at least one of the following: melanosis, leucomelanosis on at least 10% of the body, or keratosis on the hands or feet. The physicians who worked for the Joint Arsenic and Health Research Project of the Columbia University and the University of Chicago diagnosed the patients in 2007. Patients with nodular pigmentations on the hands or feet were identified as patients with keratosis. All patients were under clinical investigation due to skin lesions.

We selected 259 patients, using a systematic random-sampling method. First, we selected the first ID number from the list of patients (813 patients were in the list) as our first participant. Then, we selected patients with every third number from the list as our participants. Non-patients (n=262) were selected by matching age (±5 years) and gender with those of the patients. They were selected from the same villages where the patients were residing and had a history of drinking safe water with national standard for arsenic level (≤50 µg/L). The participants were aged 20-65 years. They were interviewed following approximately 1:1 ratio of patients and non-patients in both the sexes.

### Measurements

The Bangladeshi version of the WHOQOL Assessment-BREF (WHOQOL-BREF) questionnaire was used for evaluating the QOL. The validity and reliability of the Bangladeshi version of the WHOQOL-BREF (α=0.89) has already been tested (23,24). It consists of 24 items, covering four domains: physical condition, psychological condition, social relationships, and environmental issues (25,26). The physical domain has questions relating to daily activities, treatment compliance, pain and discomfort, sleep and rest, energy, and fatigue. The psychological domain assesses positive and negative feelings, self-esteem, body image and physical appearance, personal beliefs, and attention. The social relationship domain covers personal relationships, social support, and sexual activity. The environmental domain explores physical security, financial resources, health and social care and their availability, opportunities for acquiring new information and skills, and participation in and opportunities for recreation and transport. Besides these domains, two additional questions were used: “How satisfied are you with your life?” and “How would you rate your QOL?” The final questionnaire, thus, contained 26 items. Two items concerning an overall evaluation of QOL and satisfaction with health were not included in the calculation of the domain score and are presented separately. Each item used a 5-point Likert scale. For example: 5=Very satisfied, 4=Satisfied, 3=Neither satisfied nor dissatisfied, 2=Dissatisfied, and 1=Very dissatisfied. The higher scores indicate a better QOL. For comparing the domain scores between the patients and the non-patients, the WHOQOL-BREF scores were converted into scores from 0 to 100, with a low score of zero and a high score of 100 (24). Cronbach's α for the WHOQOL-BREF scale was 0.84 for the study.

To assess the mental health status, the self-reporting questionnaire (SRQ) was used, which was developed by the WHO (27); it is also sensitive to cultural differences. The Bangladeshi version of the SRQ has previously been used in Bangladesh (28,29). The SRQ contained 20 items, covering the major components of general mental health. All items used a 0 (no) or 1 (yes) response, with a possible total score between 0 and 20. The higher scores indicate a greater disturbance of general mental health. Cronbach's α for the SRQ was 0.81 in the study. The WHOQOL-BREF domains and the SRQ scores were calculated according to the guidelines of the WHOQOL-BREF and SRQ manuals (26,27,30).

A structured questionnaire was used for conducting face-to-face interviews during April-June 2008. Four (two male and two female) trained interviewers interviewed the participants in their homes. They have university degree and experiences of conducting field surveys and face-to-face interviews. They received a three-day training for data collection. All the participants provided verbal and written informed consents to participate in the study. The patients were interviewed in a separate room to ensure privacy. The female interviewers interviewed the female participants. The first author supervised the field work for the study. The questionnaire was pretested among 46 participants (23 patients and 23 non-patients), and necessary modifications were made based on the results.

### Statistical analysis

The basic characteristics of the participants, such as age, employment status, and annual income, were compared between the two study groups, using Student's *t*-test and chi-square analysis. Analysis of covariance (ANCOVA) was performed to compare the mean scores of WHOQOL-BREF and SRQ between the patients and the non-patients in both the sexes where age, level of education (literate/illiterate), household condition (*kaccha/pucca*), and annual income were used as covariates.

Additionally, multiple linear regression analysis was performed to identify the factors associated with the QOL. The dependent variable was the self-reported total WHOQOL-BREF score. The independent variables were age, level of education, annual income, household condition, sex, and status of the participants in terms of arsenic effects (patients or non-patients). Multicollinearity was then checked to set suitable independent variables for the regression model. Given the acknowledged existence of sociocultural differences between males and females, all analyses were performed separately for males and females, except standard multiple regression analysis. The SPSS software (version 16.0) (SPSS Inc., Chicago, IL, USA) was used for all analyses. The p values of less than 0.05 were considered significant.

### Ethical aspects

The ethical committees of the Graduate School of Medicine, The University of Tokyo (No. 2037 dated April 2008) and the Bangladesh Medical Research Council (BMRC) (BMRC/ERC/2007-2010/474 dated April 2008) approved the study protocol. The participants were informed about the study, invited to participate, and were assured of their right to decline. They gave their written informed consents before their inclusion in the study for voluntary participation.

## RESULTS

In total, 521 participants (259 arsenic patients and 262 non-patients) completed the survey; the response rate was 96.5% (521/540). Table 1 shows that the mean age of the arsenic-affected patients (n=259) was 44.9 [standard deviation (SD)±9.9] years, and the mean age of the non-patients was 43.2 (SD±9.3) years. In total, 261 participants were male, and 260 participants were female.

Of the 261 males, the mean age of 130 patients was 45.6 (SD±10.7) years, and the mean age of 131 non-patients was 46.2 (SD±9.1) years. Regarding literacy, 17.7% of the patients and 10.7% of the non-patients were illiterate while 82.3% of the patients and 89.3% of the non-patients were literate. Regarding the housing conditions, 88.5% of the patients and 67.9% of the non-patients were living in *kaccha* houses (constructed without brick) while 11.5% and 32.1% of the patients and non-patients respectively were living in *pucca* houses (constructed with brick). The difference in housing conditions was significant between the patients and the non-patients. The annual cash income was not significantly different between the patients and the non-patients in the male group.

Of the 260 females, the mean age of the patients (n=129) was 44.2 (SD±9.1) years, and the mean age of the non-patients (n=131) was 40.1 (SD±8.5) years; the difference was significant (p<0.001). Regarding literacy, 25.6% of the patients and 13.0% of the non-patients were illiterate while 74.4% of the patients and 87.0% of the non-patients were literate; the difference was significant. Regarding the housing conditions, 87.6% of the patients and 71.8% of the non-patients were living in *kaccha* houses whereas 12.4% of the patients and 28.2% of the non-patients were living in *pucca* houses. The difference in housing conditions was significant (p<0.01) between the patients and the non-patients. The mean annual cash income of the patients was US$ 761 (SD±$ 388), the mean annual cash income of the non-patients was US$ 915 (SD±$ 466), and the difference was significant (p<0.01). The annual income of the younger participants was significantly lower than that of the older participants (data not shown).

Table 2 shows the mean scores for the overall QOL, general health, WHOQOL-BREF domains, and SRQ for the patients and non-patients in both the sexes. The mean scores for the overall QOL, general health, and all WHOQOL-BREF domains were significantly lower among the patients than among the non-patients in both the sexes.

**Table. 1.** Sociodemographic data on arsenic-affected patients and non-patients (n=521)

Sociodemographic characteristics	Male			Female		
	Patients (n=130)	Non-patients (n=131)		Patients (n=129)	Non-patients (n=131)	
	n (%)	n (%)	p value	n (%)	n (%)	p value
Mean age (SD)	45.6 (10.7)	46.2 (9.1)	0.678*	44.2 (9.1)	40.1 (8.5)	<0.001*
Literacy			0.149**			0.015**
Literate	107 (82.3)	117 (89.3)		96 (74.4)	114 (87.0)	
Illiterate	23 (17.7)	14 (10.7)		33 (25.6)	17 (13.0)	
Employment			0.168**			0.621**
Yes	121 (93.1)	127 (96.9)		127 (98.4)	130 (99.2)	
No	9 (6.9)	4 (3.1)		2 (1.6)	1 (0.8)	
Housing condition			<0.001**			0.003**
*Kaccha*	115 (88.5)	89 (67.9)		113 (87.6)	94 (71.8)	
*Pucca*	15 (11.5)	42 (32.1)		16 (12.4)	37 (28.2)	
Mean annual income (SD)	$ 793 ($ 352)	$ 844 ($ 419)	0.292*	$ 761 ($ 388)	$ 915 ($ 466)	0.004*
Mean annual expenditure (SD)	$ 714 ($ 305)	$ 758 ($ 326)	0.260*	$ 693 ($ 325)	$ 801 ($ 371)	0.013*
Mean family-size (SD)	5.5 (1.9)	5.4 (1.6)	0.825*	5.2 (2.3)	5.1 (1.5)	0.868*

US$ 1=Tk 68

*p value in *t*-test and

**p value in χ^2^ analysis

*‘Kaccha’* means house built without brick and *‘Pucca’* means house built with brick

**Table. 2.** WHOQOL-BREF and SRQ scores for arsenic-affected patients and non-patients (n=521)

Condition of participants	Male					p value	Female					p value
	Patients (n=130)		Non-patients (n=131)				Patients (n=129)		Non-patients (n=131)			
	Mean	SD	Mean	SD	F		Mean	SD	Mean	SD	F	
WHOQOL-BREF												
Overall QOL	3.1	0.8	3.4	0.8	9.76	0.002	2.9	0.8	3.4	0.7	18.91	<0.001
General health	3.1	0 .9	3.6	0 .6	20.58	<0.001	3.1	0.9	3.4	0.8	9.72	0.002
Physical	55.8	16.1	61.3	11.9	10.02	0.002	51.8	13.6	58.8	11.7	18.58	<0.001
Psychological	56.8	14.4	63.7	11.4	18.45	<0.001	53.0	13.6	61.0	11.3	23.08	<0.001
Social relationships	66.9	11.3	70.2	8.2	6.75	0.010	51.5	27.8	62.8	19.4	13.82	<0.001
Environmental	59.0	11.4	61.7	9.3	4.59	0.033	52.8	10.5	59.1	9.6	24.89	<0.001
SRQ	8.4	4.4	5.2	2.9	45.42	<0.001	10.3	4.3	6.1	3.0	76.94	<0.001

ANCOVA was used with age, education level, household condition, and annual income as covariates;

WHOQOL-BREF=World Health Organization Quality of Life-BREF assessment;

SRQ=Self-reporting questionnaire (for mental health assessment);

Lower score of WHOQOL-BREF indicates low quality of life;

Higher score of SRQ indicates lower mental health status;

SD=Standard deviation

Moreover, the mean score of SRQ of the arsenic patients [mean score for male=8.4 (SD±4.4) and female=10.3 (SD±4.3)] was significantly higher than that of the non-patients [mean score for male=5.2 (SD±2.9) and female=6.1 (SD±3.0)] in both the sexes.

Table 3 shows the results of multiple linear regressions for the total WHOQOL-BREF. The factors associated with the decreased QOL score were: being male (ß=-0.26, p<0.001), effects of arsenic (patient or non-patient) (ß=0.13, p<0.01), lower age (ß=-0.18, p<0.001), and lower annual income (ß=0.15, p<0.01).

## DISCUSSION

The results of the study indicate that the overall QOL, general health, and WHOQOL-BREF domains of the arsenic-affected patients in Bangladesh were significantly poorer compared to those of the non-patients in both the sexes. A study reported that the level of QOL was lower among arsenic-affected patients than among control participants in Bangladesh (16). However, the number of patients was comparatively small (n=104), and the patients and controls were recruited from different districts (16). In our study, we selected the patients and non-patients from the same villages by matching age (±5 years) and sex. Moreover, the previous study assessed only the QOL of arsenic-affected patients but we assessed both QOL and mental health status of our participants.

One study reported that arsenic toxicity and arsenicosis have extensive social implications for the victims (31). In Bangladesh, arsenic-affected patients often face problems while seeking healthcare; they have to wait for a long time to get treatment, face discrimination in service delivery, and have inadequate access to separate facilities (32). Besides, patients with symptoms of arsenicosis suffer physical incapacity and weakness (33). Moreover, the arsenic-affected people in Bangladesh have been barred from social activities, and they often face rejection even by their immediate family members (15). For example, children of arsenic-affected people are not allowed to attend social and religious functions, and they are denied access to taking water from a neighbour's tubewell (34). Female patients are less likely to receive treatment for arsenicosis (32). These conditions might contribute to patients’ overall QOL, general health, and QOL domains. Social support is known to improve the QOL of patients with cancer (35), stigmatized diseases, such as HIV infection and AIDS, schizophrenia, and chronic diseases, such as rheumatoid arthritis (36,37). Similar interventions might be effective to improve the QOL of arsenic-affected patients.

**Table. 3.** Multiple regression analysis of WHOQOL-BREF total score (n=521)

Variable	Standardized coefficients for beta (β)	p value
Education category	0.061	0.155
Patient's status	0.131	0.002
Annual income	0.151	0.001
Age	-0.178	<0.001
Gender	-0.261	<0.001
Household condition	0.004	0.941
F value	13.26	<0.001
R2	0.13	

Standard multiple linear regression analysis;

WHOQOL-BREF=World Health Organization Quality of Life Assessment-BREF;

Patient's status (1=arsenic-affected patients and 2=non-patients), gender (1=male and 2=female), Education level (0=illiterate and 1=literate); and household condition (0=*kaccha* and 1=*pucca*)

We also quantified the mental health status of arsenic-affected patients. In our study, the SRQ score of the arsenic-affected patients was significantly lower than that of the non-patients in both the sexes. This result is consistent with a study that showed a considerably higher burden of mental health problems among people from arsenic-affected villages compared to those in arsenic-free villages in Inner Mongolia, China (17). Besides, in an arsenic-affected community in western Japan, 36% of arsenic victims (n=63) were suffering from a full or partial post-traumatic stress disorder after the arsenic poisoning in 1998 (38). In Bangladesh, the unaffected people tend to avoid and isolate arsenic-affected patients as they are generally fearful of arsenicosis (39). In consequence, some unaffected people may behave in a hostile manner, and sentiments harbour that arsenic-affected patients should either remain sequestered in their homes or leave the village (15). These conditions might have adversely affected the mental health of patients. Mental healthcare or counselling therefor is known to improve the patients’ well-being (40), and psychological interventions improve the well-being of cancer patients (41). Arsenic-affected patients might also benefit from similar interventions.

In our study, the severity of symptoms did not significantly affect the QOL and SRQ scores of the arsenic-affected patients. Moreover, the arsenic-affected patients in Bangladesh showed improvement of arsenic-induced skin lesion in the course of vitamin E and selenium intervention (42). Since all arsenic-affected patients in this study are included in the ongoing double-blind placebo-controlled trial (Bangladesh Vitamin E and Selenium trial where vitamin E and selenium or placebo are supplemented as an intervention or control group), they might have expected that their skin lesions would be reduced by the medication. However, further studies should assess the QOL and SRQ scores of arsenic-affected patients who are not taking vitamin E and selenium supplements.

In this study, being male, being arsenic-affected patients, lower age, and lower annual income were the predictors of lower QOL. In rural Bangladesh, males are typically the main earners in the family. Due to arsenicosis, some men are losing their jobs, facing difficulties in finding new jobs, and encountering social rejections (31). These conditions might have affected their QOL. Besides, chronic arsenic exposure has serious implications for arsenic-affected patients. The majority of arsenic-affected patients were considered a burden to their family and society, and they face social discrimination and marriage-related problems (31).

In our study, the younger participants showed lower QOL, which is also consistent with a study on patients receiving palliative care in Hong Kong, which showed better QOL scores among older patients (43). The younger participants in our study were more concerned about their health due to arsenicosis. Moreover, they were more likely to have lower income.

We also identified that the lower annual income was associated with lower QOL. In Bangladesh, most arsenic-affected poor patients remain untreated due to financial constraints (31). Besides, travelling a long distance and purchasing medicines are particularly difficult for poor patients in Bangladesh (32). Arsenic-affected patients in lower-income groups are more likely to face economic and social problems in Bangladesh (32). For example, 20-70% of arsenic-affected patients in Bangladesh did not receive any treatment due to financial problems (39). These circumstances might have affected their QOL.

### Limitations

The present study has three limitations. First, the study was limited by its cross-sectional design, which prohibits definitive conclusion about causality. Second, we did not measure arsenic exposure of the study participants. The arsenic exposure related measurements were done by HEALS in 2000. For this study, we only selected patients and non-patients from the database of HEALS. The patients were diagnosed by experienced physicians based on arsenic-related symptoms. Third, the patients and non-patients in this study might have had other diseases. However, the presence of other diseases might have not affected the QOL and assessment of the mental health status. Moreover, differences of the QOL and SRQ scores between the patients and non-patients are evident in our results. Despite such limitations, the results of the study provide important findings for arsenic-affected patients in Bangladesh.

### Conclusions

The results of our study revealed that both QOL and mental health status were lower among arsenic-affected patients in Bangladesh. The lower QOL scores among our study participants were associated with being arsenic-affected patients, having lower age, and having lower annual income. Our findings suggest that a mental health programme focusing on gender, physical conditions, age, and income is urgently needed for arsenic-affected patients in Bangladesh. Such a programme should aim to improve the QOL and mental health status of arsenic-affected patients. Further studies should investigate specific and practical measures.
